# Liquid Chromatography Electron Capture Dissociation Tandem Mass Spectrometry (LC-ECD-MS/MS) versus Liquid Chromatography Collision-induced Dissociation Tandem Mass Spectrometry (LC-CID-MS/MS) for the Identification of Proteins

**DOI:** 10.1016/j.jasms.2007.01.008

**Published:** 2007-05

**Authors:** Andrew J. Creese, Helen J. Cooper

**Affiliations:** School of Biosciences, University of Birmingham, Edgbaston, Birmingham, B15 2TT, United Kingdom

## Abstract

Electron capture dissociation (ECD) offers many advantages over the more traditional fragmentation techniques for the analysis of peptides and proteins, although the question remains: How suitable is ECD for incorporation within proteomic strategies for the identification of proteins? Here, we compare LC-ECD-MS/MS and LC-CID-MS/MS as techniques for the identification of proteins. Experiments were performed on a hybrid linear ion trap–Fourier transform ion cyclotron resonance mass spectrometer. Replicate analyses of a six-protein (bovine serum albumin, apo-transferrin, lysozyme, cytochrome *c*, alcohol dehydrogenase, and β-galactosidase) tryptic digest were performed and the results analyzed on the basis of overall protein sequence coverage and sequence tag lengths within individual peptides. The results show that although protein coverage was lower for LC-ECD-MS/MS than for LC-CID-MS/MS, LC-ECD-MS/MS resulted in longer peptide sequence tags, providing greater confidence in protein assignment.

For many years the established approach to protein expression and identification used two-dimensional gel electrophoresis (2D-GE). Gel electrophoresis separates mixtures of proteins and the appropriate band or spot is excised for digestion and mass spectrometric analysis [1]. Although 2D-GE is a useful analytical tool, it has limitations. For example, loading restriction limits are a major issue when expressed proteins are in low copy numbers per cell. More recently shotgun proteomics approaches have been developed in which complex mixtures of proteins are digested and analyzed by mass spectrometry [2]. Typically, protein identification involves separation of the digested peptides by liquid chromatography, followed by mass spectrometric analysis of the masses of the peptides and their sequence-specific fragments (LC MS/MS). The approach is data dependent in that the presence of peaks in the mass spectrum, which correspond to eluting peptides, trigger fragmentation of those peptide ions.

The use of online liquid chromatography coupled with mass spectrometry offers high sensitivity and improved protein identification numbers. High-performance liquid chromatography (HPLC) was first coupled with mass spectrometry in 1974 by Arpino et al. [3]. The columns had flow rates up to 2 mL/min. In 1990, online HPLC coupled with tandem mass spectrometry [4] (MS/MS, the fragmentation of selected ions) was demonstrated. The following year Huang and Henion [5] coupled online micro-LC (flow rates 3–5 μL/min) with MS/MS. Stable nanoflow rate (180 nL/min) liquid chromatography was first demonstrated in 1996 [6]. This was coupled to nanoelectrospray ionization [7, 8] by Vanhoulte in 1998 [9], thus considerably improving sensitivity.

Tandem mass spectrometry has greatly improved the confidence in protein identification. Sequences can be confirmed rather than relying on accurate mass measurement of parent ions. LC-MS/MS for proteomics typically involves collision-induced dissociation (CID) [10, 11] of the precursor peptide ions to produce sequence-specific fragment ions. CID occurs by the lowest energy pathways to produce backbone *b* and *y* ions [12]. In addition, labile post-translational modifications (PTMs) are often lost during CID.

Electron capture dissociation (ECD) [13] is a more recent fragmentation method. ECD coupled with Fourier transform ion cyclotron resonance (FT-ICR) mass spectrometry [14] has the potential to be one of the most powerful tools in proteomic studies. FT-ICR offers the highest mass accuracy and resolution of all mass spectrometry techniques, making it the obvious choice for the analysis of proteins and complex mixtures. Although ECD has been performed on ion trap mass spectrometers [15, 16, 17], comparable efficiency to that achieved with FT-ICR has not yet been demonstrated. ECD was first developed in 1998 by Zubarev et al. [13] and became a viable high-throughput method in 2001 [18] with the introduction of heated dispenser cathodes. ECD is the result of irradiation of trapped ions with low-energy electrons (∼0.2 eV). In peptide ions, the N—Cα backbone bond is cleaved, producing *c* and *z*· ions (*c*· and *z* ions are also produced as a result of hydrogen atom rearrangements [19]). After ECD, PTMs are retained on peptide backbone fragments and thus the site of modification can be assigned unambiguously. This is one of the most powerful features of ECD and has been applied to the following PTMs: γ-carboxyglutamic acid [20], phosphorylation (S, T, and Y) [21, 22], N and O glycosylation [23, 24], acylation [25], sulfation [20], methionine oxidation [26], SUMOylation [27], and ubiquitination [28]. ECD does not preferentially cleave any specific amino acid [29], and thus peptide sequence coverage is greater [30] than that for traditional fragmentation techniques such as CID. The sole exception to this is proline. The cyclic nature of the side chain means that two bonds must be cleaved to produce fragments; and this rarely occurs [31].

In the original ECD experiments, electrons were provided by a heated filament. Fragmentation took up to 30 s, a timescale unworkable for online LC analysis of complex mixtures. As mentioned above, Zubarev et al. [18] introduced an indirectly heated dispenser cathode and ECD analysis time was reduced to milliseconds. Online LC ECD MS/MS of a standard set of proteins (substance P, melittin, neurotensin, oxidized insulin chain B, and a tryptic digest of BSA) was demonstrated by Palmblad et al. [32]. ECD of all ions in the cell occurred in alternate scans. Because no ions were isolated, it was not possible to couple parent ions with fragments. The results were analyzed using the Mascot [33] database search and several sequence tags were identified. Although these were promising results, they were performed with sample amounts far greater than those available in most proteomic studies. Davidson et al. [34] demonstrated micro-HPLC ECD MS/MS of a pepsin digest of cytochrome *c*. We recently demonstrated nano-LC ECD MS/MS for the identification of the protein ROR2 isolated from human chondrocytes [35]. Zubarev and coworkers developed methods for protein identification that combine CID and ECD in single LC MS/MS experiments [36, 37, 38]. The combined approach enables important information to be derived from the relationship between *b/y* and *c/z*·, the so-called golden rules [39]; however, the approach is associated with a relatively long duty cycle.

Here, we have compared LC-CID-MS/MS and LC-ECD-MS/MS as separate proteomics techniques for the identification of proteins. Mann et al. [40] introduced the concept of the sequence tag as a measure of confidence for peptide assignment. It was suggested that a sequence tag of fewer than six amino acids would not lead to a unique match in a large protein database. We have used the parameter of a sequence tag of six consecutive cleavages [40], together with overall protein sequence coverage to compare the two approaches to protein identification. A pre-prepared six-protein tryptic digest was analyzed by use of an unmodified hybrid linear ion trap Fourier transform ion cyclotron resonance (Thermo Finnigan LTQ FT) mass spectrometer. In the ECD experiments, the mass spectrometer alternated between a high-resolution full FT-MS scan followed by two ECD scans of the two most abundant ions, doubly charged or greater. In the CID experiments, the mass spectrometer performed a high-resolution full FT-MS scan followed by three CID linear ion trap scans of the three most abundant precursor ions. Note that the aim of this study was to compare the two techniques at their individual optimal performance (in terms of number of peptide precursors producing assignable MS/MS spectra), and so experimental parameters are not identical. The data were searched against the NCBI nonredundant database using the SEQUEST algorithm within Bioworks 3.2 (Thermo Electron Corp., Bremen, Germany). The data from CID and ECD were manually checked for false positive and false negatives. The results show that *protein* sequence coverage was lower with ECD than with CID; however, ECD resulted in longer peptide sequence tags, providing greater confidence in protein assignment.

## Experimental

### Preparation of Six-Protein Mix

The six-protein tryptic digest mix (lysozyme, cytochrome *c*, yeast alcohol dehydrogenase, bovine serum albumin, apo-transferrin, and β-galactosidase) was purchased from LC Packings (Sunnyvale, CA, USA) and used without further purification. The digest was resuspended and diluted in formic acid (0.1%) (Fisher Scientific, Leicestershire, UK) to give a final concentration of 50 fmol/μL.

### LC-ECD-MS/MS and LC-CID-MS/MS

On-line liquid chromatography was performed by use of a Thermo MicroAS autosampler and Surveyor MS pump (Thermo Electron). Two microliters of the protein mix were loaded onto a 75-μm (internal diameter) Integrafrit (New Objective, Woburn, MA, USA) C_18_ column (length 10 cm) for a final loading of 100 fmol and separated over a 60-min gradient from 5 to 60% acetonitrile (J. T. Baker, Deventer, The Netherlands) (0.1% formic acid). Peptides eluted through a Picotip emitter (internal diameter 10 ± 1 μm; New Objective) directly into a Thermo Finnegan LTQ FT mass spectrometer, at approximately 300 nL/min. Each analysis (LC-ECD-MS/MS and LC-CID-MS/MS) was performed three times.

### CID-MS/MS

The mass spectrometer alternated between a full FT-MS scan (*m/z* 380–1600) and three subsequent MS/MS scans of the three most abundant precursor ions. Survey scans were acquired in the ICR cell with a resolution of 100,000 at *m/z* 400. Precursor ions were isolated and subjected to CID in the linear ion trap. Isolation width was 4 Th. Automatic gain control (AGC) was used to accumulate sufficient precursor ions (target value 1 × 10^5^ ions, maximum fill time 350 ms). CID was performed with helium gas at a normalized collision energy of 35%. The normalized collision energy is a measure of the amplitude of the resonance excitation RF voltage applied to the endcaps. The normalized collision energy scales the amplitude of the voltage to the parent mass before fragmentation. Parent ions were activated for 30 ms.

### ECD-MS/MS

The mass spectrometer alternated between a full FT-MS (*m/z* 300–1800) scan and two subsequent MS/MS scans of the two most abundant precursor ions. Survey scans were acquired in the ICR cell with a resolution of 100,000 at *m/z* 400. Precursor ions were isolated in the linear ion trap then transferred to the ICR cell for ECD. Isolation width was 4 Th. Automatic gain control was used to accumulate precursor ions in the ion trap (target value 2 × 10^5^ ions, maximum fill time 4 s). The electrons for ECD were produced by an indirectly heated barium–tungsten cylindrical dispenser cathode (5.1 mm in diameter, 154 mm from the cell, 1 mm off axis) (HeatWave Labs, Watsonville, CA, USA). The current across the electrode was approximately 1.1 A. Ions were irradiated with electrons for 50 ms at 5% energy (corresponding to 2.55 eV). Each ECD scan consisted of four coadded microscans acquired with a resolution of 50,000 at *m/z* 400.

Dynamic exclusion, which prevents reanalysis of a precursor ion, was enabled for both CID and ECD. The exclusion window was set to 5 ppm with an exclusion time of 180 s. A minimum S/N of 1000 was required before precursor ions were selected for ECD or CID. Singly-charged parent ions were not subjected to MS/MS.

Data were analyzed by use of the Xcalibur 1.4 software. Data were searched against the non-redundant NCBI database using the SEQUEST algorithm within the Bioworks 3.2 software package (Thermo Electron). Mono-isotopic precursor and fragment ions were searched with a mass tolerance of 0.01 Da (precursor) and 0.1 Da (fragment) for both ECD and CID.

Bioworks searches of ECD data consider *c* and *z*· ions only; however, because of hydrogen atom rearrangements, *c*· and *z* ions often occur in the ECD of peptides. All peptide spectra were manually searched to check for the accuracy of the database search and, in the case of ECD, the presence of *c*· and *z* ions. *c*· and *z* ions that were detected are included in all tables, figures, and results.

## Results and Discussion

**Figure 1:**
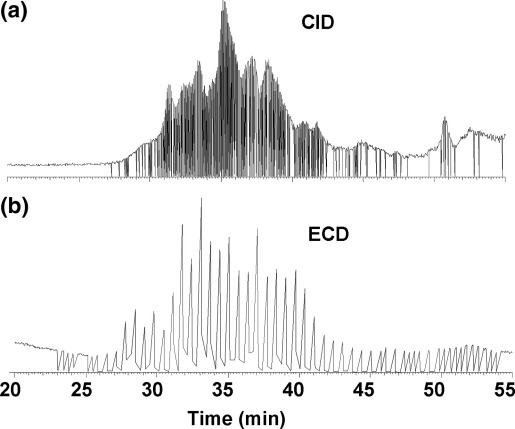
Total ion current (TIC) chromatogram obtained from an LC-CID-MS/MS (**a**) and LC-ECD-MS/MS (**b**) analysis of a tryptic digest of the six-protein mix.

Figure 1 shows the total ion current (TIC) chromatograms for an LC-ECD-MS/MS and an LC-CID-MS/MS analysis. In comparing the two techniques, we applied a practical approach in which the methods are individually optimized such that the maximum number of peptides producing interpretable MS/MS spectra were analyzed. Consequently, experimental parameters are not like for like. CID was performed in the ion trap at a rate of three MS/MS scans/s (single microscan, maximum fill time of 350 ms). ECD was performed in the ICR cell and the resulting mass spectra were the combination of four microscans with a maximum fill time of 4 s (up to 16 s per scan). Therefore over a 60-min gradient, in which peptides are eluting for about 15 min, CID can analyze over 1000 more peptides, leading to greater sequence coverage. One of the major disadvantages of the relatively long analysis time for ECD is that multiply charged peptides of lower intensity could elute without being analyzed.

**Figure 2:**
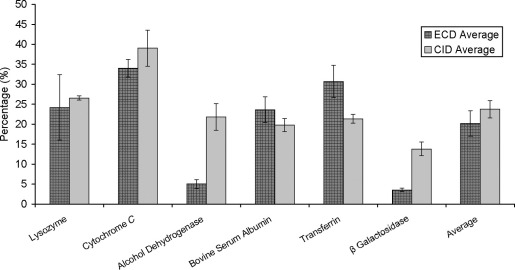
Protein sequence coverage obtained for the six proteins (averaged over three repeats) for LC-ECD-MS/MS and LC-CID-MS/MS analyses. Mean values obtained over the six proteins are also shown.

Figure 2 shows the average overall protein sequence coverage for the six proteins. The values are calculated from the Bioworks searches. It clearly shows that generally LC-CID-MS/MS results in greater sequence coverage than LC-ECD-MS/MS: 24% versus 20%, respectively. There are variations for each protein: LC-CID-MS/MS results in greater sequence coverage for lysozyme, cytochrome *c*, alcohol dehydrogenase, and β-galactosidase, whereas the reverse was true for BSA and transferrin. For lysozyme, the average of three ECD analyses gave 24%, with the CID analyses resulting in 27% coverage. For cytochrome *c*, sequence coverages of 34% (ECD) and 39% (CID) were obtained. For alcohol dehydrogenase, ECD analyses gave an average of 5% sequence coverage, with the CID analyses producing 22% coverage. The average sequence coverage obtained for BSA was 24% (ECD) and 20% (CID), for apo-transferrin was 31% (ECD) and 21% (CID), and for β-galactosidase was 4% (ECD) and 14% (CID).

**Figure 3:**
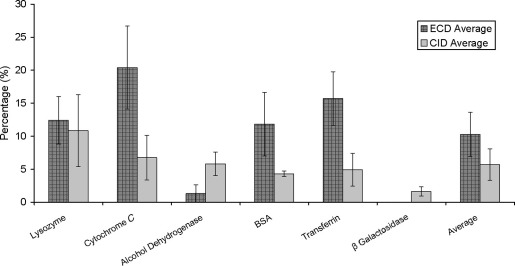
Protein sequence coverage obtained for the six proteins containing sequence tags of ≥6 consecutive amino acids (averaged over three repeats) for LC-ECD-MS/MS and LC-CID-MS/MS analyses. Mean values obtained over the six proteins are also shown.

The overall protein sequence coverage with six or more consecutive cleavages, averaged over the three replicate injections, can be seen in Figure 3. This shows that for four of the six proteins, ECD provided greater sequence coverage of the proteins when peptides contain sequence tags ≥6 amino acids long. The average sequence coverages obtained using these criteria were: lysozyme 12.4% (ECD) and 10.8% (CID); cytochrome *c* 20.4% (ECD) and 6.7% (CID); alcohol dehydrogenase 1.3% (ECD) and 5.8% (CID); BSA 11.85 (ECD) and 4.3% (CID); apo-transferrin 15.7% (ECD) and 4.9% (CID); and β-galactosidase 0% (ECD) and 1.6% (CID).

**Figure 4:**
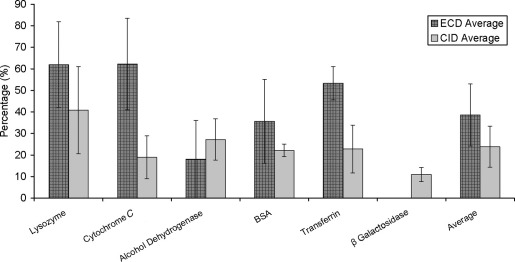
Proportion of peptides identified containing sequence tags of ≥6 consecutive amino acids for the six proteins (averaged over three repeats) for LC-ECD-MS/MS and LC-CID-MS/MS. Mean values obtained over the six proteins are also shown.

Figure 4 shows the percentage of peptides detected for each analysis type that had ≥6 amino acid sequence tags. An average of 62.0% of the lysozyme peptides detected in the LC-ECD-MS/MS analyses had sequence tags of ≥6 amino acids compared with 40.8% of those detected in the LC-CID-MS/MS analyses. The results for the remaining proteins were: cytochrome *c* 62.2% (ECD) and 18.9% (CID); alcohol dehydrogenase 18% (ECD) and 27.2% (CID); BSA 35.6% (ECD) and 22.2% (CID); apo-transferrin 53.3% (ECD) and 22.9% (CID); and β-galactosidase 0% (ECD) and 11.0% (CID). These results suggest that LC-ECD-MS/MS generally produces longer and therefore more reliable sequence tags than LC-CID-MS/MS. When all six proteins are considered, the ECD analyses resulted in 38.5% coverage, whereas the CID analyses gave 23.8% coverage.

Table 1 shows the peptides that were detected in all six analyses along with the average total number of cleavages, the average number of consecutive cleavages, and the mass accuracy of the fragments. [The ECD fragment mass accuracy is not as high as expected in some cases. Mass calibration parameters are based on the number of ions in the ICR cell as set by the automatic gain control (AGC). If the AGC target is not reached, incorrect calibration parameters will be applied. Nevertheless, all ECD fragments were observed within 10 ppm mass accuracy. Note also that ECD has a far greater mass accuracy than that of CID. This is to be expected because ECD was detected in the ICR cell and CID in the linear ion trap.] Table 1 shows that ECD cleaved more bonds overall than CID and that ECD produces longer-sequence tags than CID. Seven of the 12 peptides had ECD-derived sequence tags of ≥6 consecutive cleavages. Alcohol dehydrogenase peptide [VVGLSTLPEIYEK] fell below this threshold as a result of the central proline residue. ECD rarely fragments the peptide backbone at proline as a result of the residue’s cyclic nature [31]. It has been argued that the lack of cleavage of proline does not preclude sequence determination: A mass difference between consecutive fragments corresponding to amino acids Xxx and Pro implies the sequence must be Xxx-Pro rather than Pro-Xxx because it is N-terminal proline cleavage, which does not occur. That argument has not been applied in the current study and the numbers calculated are absolute. For CID, four of the 12 peptides had sequence tags of ≥6 consecutive cleavages.

**Table 1:** Sequence tags obtained for those peptides observed in all analyses^a^

Protein	Peptide	ECD			CID		
		Sequence tag		Mass accuracy (ppm)	Sequence tag		Mass accuracy (ppm)
		Abs	Consec		Abs	Consec	
Cytochrome *c*	TGPNLHGLFGR	8.0	8.0	4.5	8.3	8.3	84.5
Cytochrome *c*	EDLIAYLK	5.0	4.3	8.1	4.0	4.0	125.7
Alcohol dehydrogenase	VVGLSTLPEIYEK^b^	8.7	4.3	4.7	6.0	4.0	102.3
BSA	YLYEIAR	5.0	4.7	1.7	5.3	4.7	147.8
BSA	HLVDEPQNLIK^b^	9.0	4.0	7.1	6.0	3.3	107.6
BSA	LVNELTEFAK	9.0	9.0	4.2	7.3	7.3	56.3
BSA	LGEYGFQNALIVR	8.7	7.0	3.4	7.0	5.3	72.8
Apo-transferrin	ELPDPQESIQR^b^	6.7	5.0	2.2	7.3	5.3	75.2
Apo-transferrin	DNPQTHYYAVAVVK	10.7	10.7	4.8	7.0	4.7	96.9
Apo-transferrin	DKPDNFQLFQSPHGK^b^	9.3	8.0	9.1	8.7	5.3	70.9
Apo-transferrin	TYDSYLGDDYVR	9.0	8.0	3.1	7.3	7.0	87.5
Apo-transferrin	HSTVFDNLPNPEDR^b^	10.7	7.0	4.0	9.0	7.7	62.4
	Average			4.7			90.8

a Abs, total number (mean over three replicates) of N-Cα (ECD) or N-C_o_ (CID) cleavages observed; Consec, number (mean over three replicates) of consecutive N-Cα (ECD) or N-C_o_ (CID) cleavages observed. Mass accuracy of fragments is given.

b Proline limited coverage.

**Figure 5:**
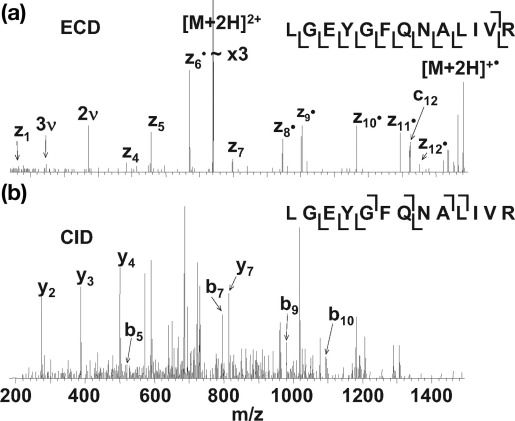
ECD (**a**) and CID (**b**) mass spectra of the doubly protonated ions of tryptic peptide [421–433] LGEYGFQNALIVR from bovine serum albumin (υ denotes harmonic).

**Figure 6:**
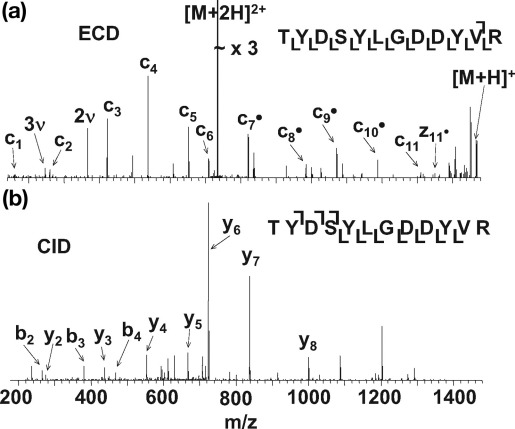
ECD (**a**) and CID (**b**) mass spectra of doubly protonated ions of tryptic peptide [671–682] TYDSYLGDDYVR from apo-transferrin (υ denotes harmonic).

**Figure 7:**
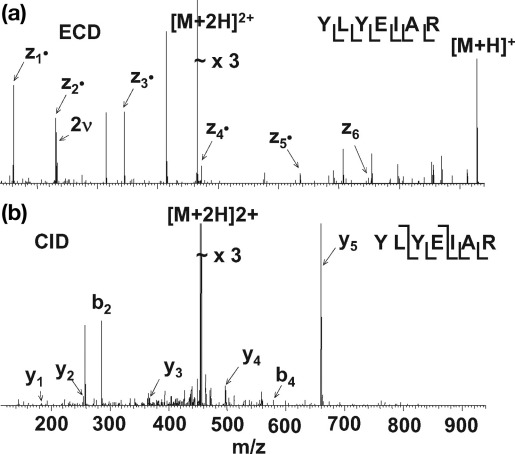
ECD (**a**) and CID (**b**) mass spectra of doubly protonated ions of tryptic peptide [161–167] YLYEIAR from bovine serum albumin (υ denotes harmonic).

Figure 5, Figure 6, Figure 7 show LC-ECD-MS/MS and LC-CID-MS/MS mass spectra for three different peptides. The ECD mass spectrum for the bovine serum albumin peptide [LGEYGFQNALIVR] [M+2H]^2+^ ions (Figure 5a) shows 83% peptide coverage. Ten of the 12 N—Cα bonds were cleaved, eight of which were consecutive. This is a reliable sequence tag. Figure 5b shows the CID mass spectrum for the same species, showing 58% peptide coverage with seven of 12 peptide bonds cleaved; however, only four of these are consecutive. Figure 6a shows the ECD mass spectrum of peptide [TYDSYLGDDYVR] [M+2H]^2+^ ions from apo-transferrin. The ECD mass spectrum gave 100% peptide coverage: eleven of 11 N—Cα bonds were cleaved, producing a complete sequence tag. The CID mass spectrum of the same peptide (Figure 6b) revealed nine of 11 peptide bonds cleaved, leading to 82% peptide coverage. The nine cleavages are consecutive and thus a reliable sequence tag was obtained. The ECD mass spectrum of [YLYEIAR] [M+2H]^2+^ ions of bovine serum albumin (Figure 7a) revealed six of 6 N—Cα bonds were cleaved, giving 100% peptide coverage. The CID spectrum for the same peptide resulted in five of six peptide bonds being cleaved with 83% peptide coverage.

## Conclusion

The results show that for the six-protein tryptic digest LC-CID-MS/MS results in greater overall protein coverage than LC-ECD-MS/MS. This observation appears to be the result of the greater speed of analysis of LC-CID-MS/MS. However, LC-ECD-MS/MS analysis produces more accurate and, on average, longer peptide sequence tags (consecutive cleavages) than LC-CID-MS/MS. The protein sequence coverage containing sequence tags of ≥6 consecutive amino acids was greater for LC-ECD-MS/MS than for its counterpart. The proportion of peptides producing tags of ≥6 consecutive amino acids was greater for ECD analyses than for CID. Twelve peptides were observed in all analyses; ECD produced longer sequence tags than CID for eight of these, seven of which had tags of six cleavages or more. The CID results show that only four of the peptides had tags of six or more consecutive cleavages.

Note that assignment of ECD mass spectra involved manual inspection to identify hydrogen atom rearrangements (*c*· and *z* ions). This is executable for small datasets, although improved software for automated ECD analysis is required for application to large datasets.

This work has taken advantage of the hybrid nature of our instrument—that is, the presence of both an ion trap and ICR cell. CID experiments were performed in the ion trap and the findings cannot therefore be extrapolated to analyses in which CID is performed in the ion trap but detected in the ICR cell, or performed and detected in the ICR cell. Those methods are associated with longer duty cycles and thus fewer peptides would be analyzed and protein sequence coverage would be reduced.

In summary, the results suggest that LC-ECD-MS/MS offers improved confidence in protein identification as a consequence of peptide sequence coverage—that is, sequence tag length. However, overall protein coverage is lower than that for LC-CID-MS/MS. The latter needs to be counterbalanced by improved speed of analysis for LC-ECD-MS/MS. Improved speed of analysis for LC-ECD-MS/MS is also vital for proteomic studies in which many peptides must be sampled to maximize proteome coverage, that is, the number of proteins identified. Nevertheless, LC-ECD-MS/MS does offer great potential for confident protein identification.
